# Endoplasmic Reticulum Stress and Impairment of Ribosome Biogenesis Mediate the Apoptosis Induced by *Ocimum* x *africanum* Essential Oil in a Human Gastric Cancer Cell Line

**DOI:** 10.3390/medicina58060799

**Published:** 2022-06-14

**Authors:** Wongwarut Boonyanugomol, Kamolchanok Rukseree, Pornpan Prapatpong, Onrapak Reamtong, Seung-Chul Baik, Myunghwan Jung, Min-Kyoung Shin, Hyung-Lyun Kang, Woo-Kon Lee

**Affiliations:** 1 Department of Sciences and Liberal Arts, Amnatcharoen Campus, Mahidol University, Amnatcharoen 37000, Thailand; kamolchanok.ruk@mahidol.edu; 2 Department of Public Health, Amnatcharoen Campus, Mahidol University, Amnatcharoen 37000, Thailand; pornpan.pra@mahidol.edu; 3 Department of Molecular Tropical Medicine and Genetics, Faculty of Tropical Medicine, Mahidol University, Bangkok 10400, Thailand; onrapak.rea@mahidol.edu; 4 Department of Microbiology and Convergence Medical Science, College of Medicine, Gyeongsang National University, Jinju 52727, Korea; scbaik@gnu.ac.kr (S.-C.B.); mjung@gnu.ac.kr (M.J.); mkshin@gnu.ac.kr (M.-K.S.); kangssi@gnu.ac.kr (H.-L.K.); wklee@gnu.ac.kr (W.-K.L.)

**Keywords:** *Ocimum* x *africanum*, essential oil, AGS gastric cancer, ER stress, ribosome biogenesis, apoptosis

## Abstract

*Background and Objectives*: Gastric cancer remains a major unmet clinical problem worldwide. Although conventional medical treatments are available, their curative effects are generally unsatisfactory. Consequently, it remains necessary to search natural products for potential alternatives in treating gastric cancer patients. *Ocimum* x *africanum* Lour. is a culinary herb that has been used in folk medicine for various diseases, but little is known regarding its anti-cancer activity against gastric cancer cells. In the current study, we focus on the anti-cancer mechanisms of *O.* x *africanum* essential oil (OAEO) in the AGS human gastric cancer cell line. *Materials and Methods*: After OAEO treatment, AGS cell viability was evaluated by MTT assay. Cell migration and apoptotic nuclear morphology were determined by wound-healing assay and DAPI staining, respectively. Gene expression levels of apoptosis-related genes were quantified by qRT–PCR. Differential protein expression was determined with an LC–MS/MS-based proteomics approach to identify the key proteins that may be important in the anti-cancer mechanisms of OAEO on AGS cells. The chemical constituents of OAEO were identified by GC–MS analysis. *Results*: We found OAEO to exhibit a potent growth-inhibiting effect on AGS cells, with an IC_50_ value of 42.73 µg/mL. After OAEO treatment for 24 h, AGS cell migration was significantly decreased relative to the untreated control. OAEO-treated AGS cells exhibited common features of apoptotic cell death, including cell shrinkage, membrane blebbing, chromatin condensation, and nuclear fragmentation. Apoptotic cell death was confirmed by qRT–PCR for apoptosis-related genes, revealing that OAEO decreased the expression of anti-apoptotic genes (*BCL2* and *BCL-xL*) and activated pro-apoptotic genes and apoptotic caspase genes (*TP53*, *BAX*, *CASP9*, *CASP12*, and *CASP3*). Moreover, expression of *CASP8* was not changed after treatment. Proteomic analysis revealed that OAEO may produce a signature effect on protein clusters relating to unfolded protein accumulation, thereby inducing severe ER stress and also impairing ribosome synthesis. STRING analysis revealed seven up-regulated and 11 down-regulated proteins, which were significantly associated with protein folding and ribosome biogenesis, respectively. Using GC–MS analysis, 6-methyl-5-hepten-2-one, citral, neral, and linalool were found to be the major chemical constituents in OAEO. *Conclusions*: Taken together, these results indicate that OAEO has a potential anti-proliferative effect on AGS cells. Our molecular findings show evidence supporting an important role of ER stress and ribosome biogenesis impairment in mediating the induction of cell death by OAEO through the mitochondrial-apoptotic pathway. This study, therefore, provides fundamental knowledge for future applications using OAEO as an alternative therapy in gastric cancer management.

## 1. Introduction

Chronic infection by *Helicobacter pylori* significantly increases the risk of developing gastric cancer (GC) [1]. Surgical resection is a conventional therapy routinely used to treat GC; however, once a case has achieved the metastatic stage, patient prognosis is poor, with median survival of only around one year [2]. Chemotherapeutic drugs are another typical treatment modality, but they afflict patients with systemic side effects such as nausea, vomiting, anorexia, diarrhea, oral mucositis, and numbness [3]; in addition, chemotherapeutic resistance, which might lead to treatment failure, has been reported in gastric cancer cells [4]. Given the issues with these established therapeutic approaches, it is imperative to investigate agents from natural products for their effectiveness in suppressing cancer cells, which may lead to the development of complementary or alternative agents that improve GC treatment in the future.

Among sources of natural products, plants are considered remarkable potential reservoirs of biologically active compounds that elicit many beneficial health effects, and, hence, many drugs have been developed from compounds of plant origin [5]. As sources of phytochemicals, essential oils are of particular interest; these mixtures of volatile compounds contain various plant secondary metabolites and have long been used in traditional medicine. An increasing number of published studies concerning essential oils have emerged over the past two decades, reflecting the interesting potential of some constituent compounds for pharmaceutical uses [6]. Indeed, essential oils or their constituents have demonstrated multiple cancer-preventive effects involving apoptosis, cell cycle arrest, anti-metastatic activity, anti-angiogenic activity, or induction of cell death through increased generation of intracellular reactive oxygen species/reactive nitrogen species [7]. The genus *Ocimum*, which belongs to the family Lamiaceae and is widely distributed in subtropical regions such as Asia, Africa, and South America, encompasses some of the most popular medicinal herbs, including a species often called the king of herbs, although the most common use of these species is as culinary ingredients [8]. The essential oils derived from *Ocimum* species have demonstrated many biologically active constituents with important biological properties such as insecticidal, anti-oxidant, anti-lipidemic, anti-cancerous, anti-inflammatory, and anti-microbial activities [9].

Within this genus, *Ocimum* x *africanum* Lour. or lemon basil (synonym *Ocimum americanum*, *Ocimum* x *citriodorum*) is an annual or short-lived perennial herb that grows 45–105 cm tall and has white flowers and green leaves with slightly serrated edges. In Thailand, this plant is usually called *maenglak*; it is widely cultivated in many areas and available for purchase in local markets. Along with several other types of basil, it is a culinary herb widely used in Thai cuisine, including in curries, soups, noodle dishes, and steamed or grilled dishes [10]. This plant has also been used as traditional medicine for many conditions, such as cough and headache, and for its expectorant, anti-flatulence, anti-fungal, and anti-emetic effects [10]. Previously, the essential oil extracted from this plant (*O. americanum*) has been reported to demonstrate in vitro anti-microbial activity, specifically inhibiting the growth and disrupting the biofilms of oral pathogens, including *Streptococcus mutans* and *Candida albicans* [11]. Our previous study found that the essential oil extracted from another plant in the same genus, *O. tenuiflorum*, termed Holy basil or *kapraw* (in Thai), exhibited in vitro anti-cancer activity against a human gastric cancer cell line (AGS) by means of inducing apoptosis through up-regulation of pro-apoptotic and caspase-encoding genes [12]. It remains unknown whether *O.* x *africanum* essential oil exhibits similar in vitro anti-cancer activity against human gastric cancer cells, along with the underlying molecular mechanism of such an effect. Proteomic technologies in conjunction with advanced bioinformatics are useful for identifying and quantifying abundant cellular proteins, ultimately leading to a deep understanding of how the proteome changes in response to stimuli [13,14]. The main objective of the present study is to determine the ability of *O.* x *africanum* essential oil (OAEO) to inhibit the in vitro growth and survival of the human gastric cancer AGS cell line and to analyze the possible mechanisms of its action; towards this end, we applied quantitative reverse transcription–polymerase chain reaction (qRT–PCR) and liquid chromatography with tandem mass spectrometry (LC–MS/MS)-based proteomics.

## 2. Materials and Methods

### 2.1. Plant Material and Essential Oil Preparation

**Figure 1:**
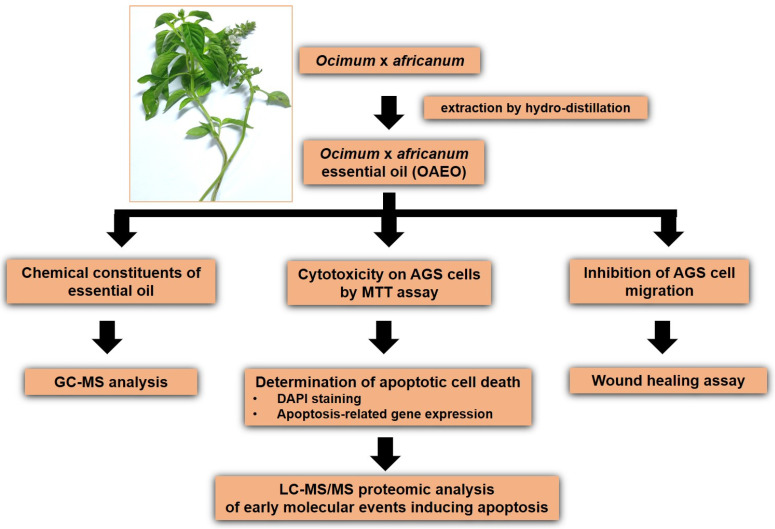
Flow diagram of this study.

A fresh plant of *O.* x *africanum* was purchased from the local market of Amnatcharoen province in northeast Thailand. The fresh leaves were washed and essential oil subsequently extracted by hydro-distillation according to our previous report [12]. The obtained yellow essential oil was kept in a small brown bottle, tightly sealed, and stored at −20 °C for further experiments. Stock OAEO solution was prepared by solubilizing with absolute ethanol. Botanical identification of the plant was performed by a botanist, and a voucher herbarium specimen was deposited at the official herbarium of the Pharmaceutical Botany Department, Mahidol University (PBM 005564). A flow diagram of the experiments is shown in Figure 1.

### 2.2. AGS Cell Culture

The human gastric cancer AGS cell line (ATCC CRL-1739) was obtained from the Department of Microbiology and Convergence Medical Science, College of Medicine, Gyeongsang National University, Republic of Korea. This cell line was grown as a monolayer at 37 °C under a humidified 5% CO_2_ atmosphere in RPMI 1640 medium (Gibco, Life Technologies Corp., Grand Island, New York, NY, USA) containing 10% fetal bovine serum (FBS) (Gibco, Life Technologies Ltd., Paisley, OR, UK) and antibiotics (100 µg/mL of streptomycin and 100 U/mL of penicillin) (Gibco, Life Technologies Corp., Grand Island, New York, NY, USA).

### 2.3. AGS Cell Viability by MTT Assay

In order to measure AGS cell viability, the MTT (3-(4, 5-dimethylthiazolyl-2)-2, 5-diphenyltetrazolium bromide) assay was performed. Briefly, AGS cells were seeded in 96-well plates at a density of 1.5 × 10^4^ cells/well and incubated at 37 °C (5% CO_2_) for 24 h. Then, the cells were treated with different concentrations of OAEO and 5-fluorouracil (5-FU). Following 24 h incubation, the culture media was discarded, and viable cells were quantified by incubating with MTT solution (5 mg/mL) for 4 h. The purple color of precipitated formazan was solubilized by dimethyl sulfoxide (DMSO), and its absorbance was measured by a microplate reader at 570 nm (SPECTROstar NANO, BMG Labtech, Ortenberg, Germany). The percentage of viable cells was compared against vehicle-treated control cells (0.5% ethanol). All experiments were performed in triplicate, and the 50% inhibitory concentration (IC_50_) of OAEO was calculated using an AAT Bioquest IC_50_ calculator (AAT Bioquest, Inc., Sunnyvale, CA, USA).

### 2.4. Cell Migration Assay

AGS cells were cultured in a 12-well plate (1.5 × 10^5^ cells/well) for 24 h to achieve monolayer confluence. Then, the cells were scratched at the well center using a 200 µL pipette tip and subsequently washed with 1X PBS to remove cell debris. Cells were then cultured in serum-free media containing OAEO (IC_50_) for 24 h; vehicle control cells were cultured in serum-free media without OAEO. Image acquisition was performed with an inverted microscope (ECLIPSE Ts2-FL, Nikon, Tokyo, Japan), and wound area width was measured by NIS Elements Imaging Software version 4.60 (Nikon, Tokyo, Japan). The percentage of wound closure was quantified by comparing the remaining cell-free area (at 24 h) with the initial wound area (at 0 h).

### 2.5. AGS Nuclear Morphology by DAPI Staining

AGS cells were treated with OAEO at the determined IC_50_ value for 6, 12, and 24 h. The treated and corresponding vehicle control cells were washed with 1X PBS and fixed with absolute ethanol for 10 min. Then, the fixed cells were washed with 1X PBS and stained with 2.5 µg/mL DAPI solution (4′,6-diamidino-2-phenylindole dihydrochloride) (Sigma-Aldrich, St. Louis, MO, USA) for 15 min in the dark. The nuclear morphology of stained cells was observed through a fluorescence microscope (BX53F2, Olympus, Tokyo, Japan). All determinations were performed in triplicate as independent experiments.

### 2.6. Expression Levels of Apoptosis-Related Genes by qRT–PCR

AGS cells were cultured in 6-well plates at a cell density of 3 × 10^5^ cells/well and incubated at 37 °C. After 24 h, the cells were treated with OAEO at the IC_50_ concentration for 6, 12, and 24 h. Cells cultured in media without OAEO were used as vehicle controls. At the end of the exposure period, total RNA was extracted using Ribozol^TM^ RNA Extraction Reagent (Amresco, VWR, Solon, OH, USA) in accordance with the manufacturer’s instructions. The concentration of isolated RNA was determined using a UV–vis spectrophotometer (NanoDrop^TM^ One^c^, Thermo Fisher Scientific, Waltham, MA, USA). One microgram of RNA from each sample was utilized for cDNA synthesis using a cDNA synthesis kit (Vivantis, Malaysia) according to the manufacturer’s protocol. Gene expression was examined by qRT–PCR using previously reported primer sequences (Table 1) (Figure S1) [15,16,17,18,19,20,21,22]. Each PCR reaction was prepared in a total volume of 20 µL, consisting of 1X SYBR Green Realtime PCR master mix (TOYOBO, Osaka, Japan), 0.5 µM primers, and 1 µL cDNA. PCR amplification was performed in a LightCycler^®^ 96 real-time PCR instrument (Roche Diagnostics GmbH, Mannheim, Germany) with the following program: 95 °C for 10 min, then 40 cycles of 95 °C for 30 s, 55 or 60 °C for 30 s, and 72 °C for 30 s. Cycle threshold (Ct) values for each gene were normalized to a housekeeping gene (*GAPDH*), and relative gene expression levels were quantified by 2^−^^△△CT^ analysis with comparison to the control group.

### 2.7. Differential Protein Analysis by LC–MS/MS

AGS cells were seeded into cell culture dishes at a density of 8 × 10^5^ cells/dish. After 24 h incubation, culture media were removed and the cells treated with OAEO (IC_50_ value). At 6 h of incubation, AGS morphological changes began to appear in the treated cells. At that time, total protein was extracted from treated and control cells using RIPA lysis and extraction buffer containing a Halt^TM^ protease inhibitor cocktail (Thermo, Rockford, IL, USA). The cell extracts were centrifuged at 12,000 rpm for 15 min to remove debris, and the supernatants were collected. Protein concentration was determined by the Bradford technique. Next, lysate proteins were separated on a 12% SDS-PAGE gel and stained with Coomasie Brilliant Blue G250 solution (Bio-Rad, Hercules, CA, USA). Gel slices were then destained till colorless in 50% acetonitrile (ACN, Sigma-Aldrich) in 50 mM ammonium bicarbonate (Sigma-Aldrich). Following destaining, gel fragments were incubated at 60 °C in 4 mM DL-dithiothreitol (DTT, Sigma-Aldrich) for 15 min. The embedded proteins were next alkylated with 250 mM iodoacetamide (Sigma-Aldrich) and incubated for 30 min at room temperature in the dark. After removing all solution, the gel pieces were dehydrated in 100% ACN, rehydrated with 10 ng/L trypsin in 50 mM ammonium bicarbonate, and incubated at 37 °C overnight to break down the proteins. The resulting peptides were retrieved with ACN, and the supernatant was collected and dried by vacuum centrifugation (TOMY, Tokyo, Japan). To conduct LC–MS/MS analysis, the dried peptides were resuspended in 0.1% formic acid.

For the LC–MS/MS method, a MicroTOF Q II mass spectrometer was employed in conjunction with an UltimateTM 3000 nano-LC system. The column used was an Acclaim PepMap RSLC 75 m × 15 cm nanoviper C18 column with a particle size of 2 m and a pore size of 100 angstrom (Thermo Scientific, Waltham, MA, USA). For peptide identification, mass spectrometry data were analyzed with the MASCOT search engine 2.3 (Matrix Science, Ltd., London, UK) and compared against the Swiss-Prot database. The following search parameters were used: one missed cleavage, trypsin digestion, 0.8 Da peptide tolerance, 0.8 fragment mass tolerance, carbamidomethyl (C) and oxidation (M) variable modifications, and organism *Homo sapiens*. The significance level was set at 0.05. Protein abundance was calculated using the exponentially modified protein abundance index (emPAI). The STRING database was used for pathway analysis of differentially abundant proteins.

### 2.8. Analysis of OAEO Chemical Constituents by GC–MS

The volatile chemical composition of OAEO was determined through gas chromatography–mass spectrometry analysis (GC–MS) in conjunction with a PerkinElmer Headspace Therbo matrix 40 auto-sampler (Clarus 690, PerkinElmer, Waltham, MA, USA) according to our previous report [12]. A 30 m × 0.25 mm i.d. Elite-5MS capillary column with 0.25 µm film thickness (Perkin Elmer, Waltham, MA, USA) was used for GC separation. The OAEO sample was placed in a 22 mL headspace vial and heated for 10 min at 90 °C (equilibrium temperature). The injection time was 0.10 min with constant mode.

The separation of the OAEO sample was performed using the GC condition as follows. The injector was maintained at 280 °C in a split mode (10:1). The column oven temperature was initially set at 60 °C for 1 min and then increased by 4 °C/min until 280 °C, where it was held at this temperature for 5 min. The carrier gas was helium with a GC grade and a set flow rate of 1 mL/min constantly. MS detection was carried out at 200 °C, and the electron impact (EI) mode was required, using the full scan mode from *m*/*z* 30 to 600 with a scanning speed at a low level. Volatile chemicals were identified based on a comparison of corresponding GC retention times and mass spectra to a reference from the US National Institute of Standard and Technology (NIST 2017), with more than 75% similarity being required for a match.

### 2.9. Statistical Analysis

Data are presented as mean ± standard deviation. The statistical significance of differences between treatment and control groups was determined by Student’s *t*-test, with *p*-values less than 0.05 considered to indicate a significant difference.

## 3. Results

### 3.1. Inhibition of AGS Cell Viability by OAEO

**Figure 2:**
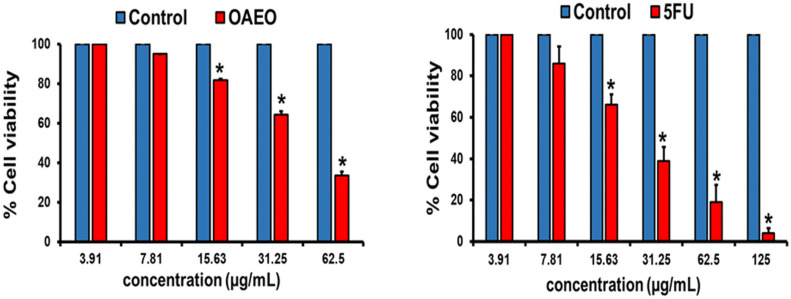
Inhibition of cell viability by OAEO and 5-FU in AGS cells, as determined by MTT assay. Data are presented as the mean ± standard deviation of three independent experiments. * *p* < 0.05 vs. control.

In order to determine whether OAEO treatment inhibits AGS cell viability, cells were co-cultured with a range of OAEO concentrations for 24 h. Subsequent MTT assays revealed that viability was significantly decreased after OAEO exposure (Figure 2). The 50% inhibitory concentration (IC_50_) of OAEO in AGS cells was found to be approximately 42.73 µg/mL. In this study, 5-FU was used as the reference chemotherapeutic drug, and its IC_50_ value was evaluated to be 22.51 µg/mL.

### 3.2. Inhibition of AGS Cell Migration by OAEO

**Figure 3:**
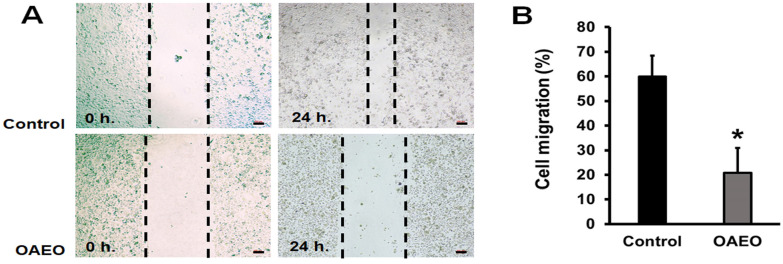
Effect of OAEO on AGS cell migration as determined by in vitro wound-healing assay. (**A**) Representative images of the initial wound area (0 h) and the remaining cell-free area (24 h) in OAEO-treated and vehicle control cultures. (**B**) Rates of cell migration (%) were quantified by comparing the wound area before and after treatment. Data are presented as mean ± standard deviation from triplicate experiments. * *p* < 0.05 vs the vehicle control group.

Wound-healing assays were used to evaluate the effect of OAEO on the migratory activity of AGS cells. The results are shown in Figure 3. After treatment for 24 h, cell migration of OAEO-treated AGS cells was significantly decreased (at 20.8%) relative to the vehicle control (at 59.9%).

### 3.3. Morphological Features of Cell Death

**Figure 4:**
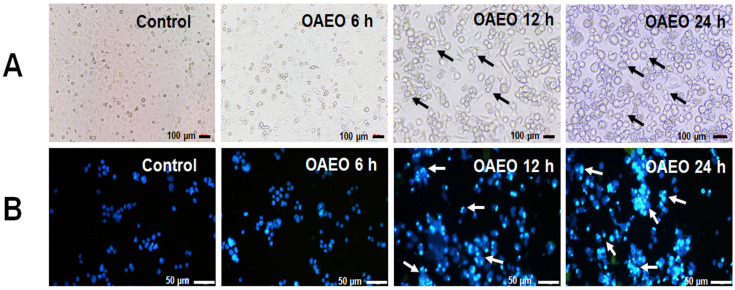
(**A**) Morphology of vehicle control and OAEO-treated AGS cells at 6, 12, and 24 h, as observed by inverted microscope. (**B**) Nuclear morphology of vehicle control and OAEO-treated AGS cells at 6, 12, and 24 h, as visualized by DAPI staining. All determinations were performed in triplicate as independent experiments.

At 6 h, cell morphological changes began to appear in the treated group. After 12–24 h of treatment, apoptotic cell morphologies were observed more apparently in AGS cells treated with OAEO (IC_50_ value) when observed by inverted microscope, including cell shrinkage and membrane blebbing (black arrows) (Figure 4A). As shown in Figure 4B, DAPI staining and fluorescence microscopy revealed typical patterns of chromatin condensation and fragmentation in AGS-treated cells (white arrows).

### 3.4. Expression Levels of Apoptosis-Related Genes in OAEO-Treated AGS Cells

**Figure 5:**
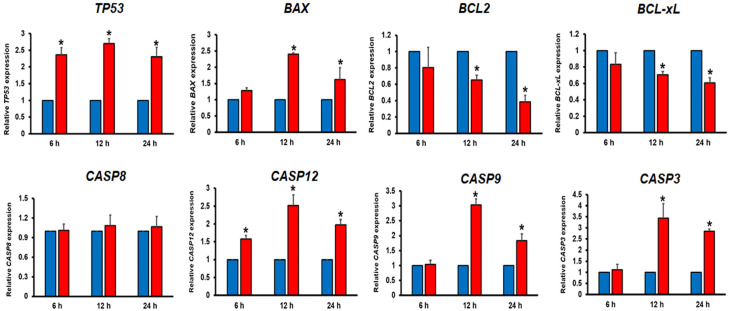
Effects of OAEO on apoptosis-related gene expression in AGS-treated cells. Expression levels of *TP53*, *BAX*, *BCL2*, *BCL-xL*, *CASP8*, *CASP12*, *CASP9*, and *CASP3* were determined by qRT–PCR at 6, 12, and 24 h (blue bars: untreated control; red bars: OAEO treatment). Relative gene expression data are presented as mean ± SD. * *p* < 0.05 vs. vehicle control cells.

In light of OAEO inducing apoptosis in AGS cells, the expression of apoptosis-related genes was investigated by qRT–PCR at 6, 12, and 24 h (Figure 5). After 6–24 h of treatment, the expression levels of *TP53* (2.4-fold at 6 h; 2.7-fold at 12 h; 2.3-fold at 24 h) and *CASP12* (1.6-fold at 6 h; 2.5-fold at 12 h; 2.0-fold at 24 h) were significantly up-regulated in OAEO-treated AGS cells. After 12–24 h of treatment, the expression levels of *BAX* (2.4-fold at 12 h and 1.6-fold at 24 h), *CASP9* (3.0-fold at 12 h and 1.8-fold at 24 h), and *CASP3* (3.4-fold at 12 h and 2.8-fold at 24 h) were significantly increased. Meanwhile, levels of the anti-apoptotic genes *BCL2* (0.7-fold at 12 h and 0.4-fold at 24 h) and *BCL-xL* (0.7-fold at 12 h and 0.6-fold at 24 h) were significantly decreased in AGS-treated cells. However, we found that the expression levels of *CASP8* were not changed.

### 3.5. Differential Protein Expression of OAEO-Treated AGS Cells by LC–MS/MS

At 6 h of OAEO treatment, we found that morphological changes began to appear in the treated cells. In order to understand the biological relevance of differential protein expression in response to OAEO treatment of AGS cells, cell proteomes were profiled by LC–MS/MS. A total of 4398 proteins were detected in both control and treated cells, with treated cells having 43 and 25 proteins, respectively, up-regulated and down-regulated (by more than 2-fold). Table 2 lists the most differentially expressed proteins. The top five up-regulated proteins were 78 kDa glucose-regulated protein, 60 kDa heat shock protein, heat shock 70 kDa protein 1A/1B, filamin-B, and heat shock 70 kDa protein 6. Meanwhile, the top five down-regulated proteins were 40S ribosomal protein S5, transgelin-2, 60S ribosomal protein L31, peroxiredoxin-2, and triosephosphate isomerase. The list of up-regulated and down-regulated proteins can be found in the Supplementary file (Tables S2 and S3).

### 3.6. Protein–Protein Interaction Network of the Identified Proteins

**Figure 6:**
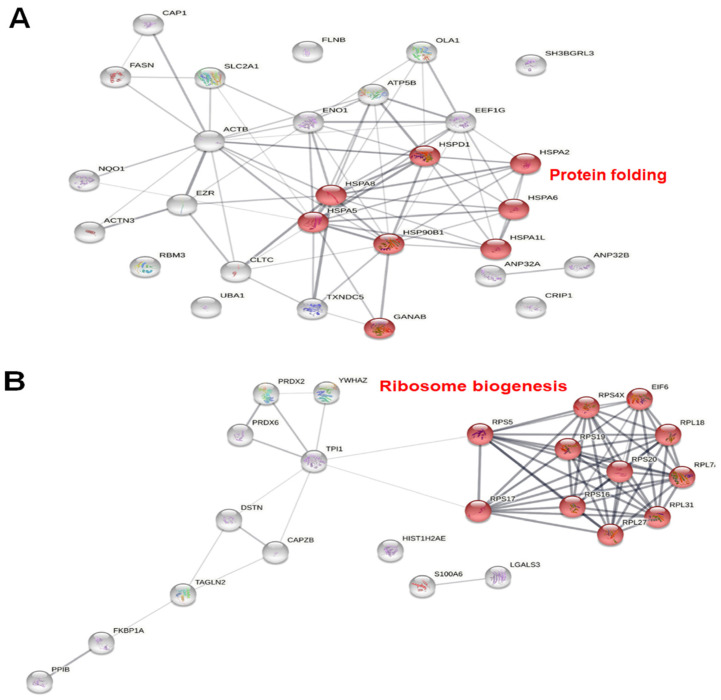
Protein–protein interaction networks for up-regulated (**A**) and down-regulated (**B**) proteins according to the STRING database.

Predicted protein interaction networks were constructed for differentially expressed proteins using the STRING database. Figure 6A depicts the resulting network for up-regulated proteins, of which eight are significantly associated with protein folding, including GRP78 (*HSPA5*) and several heat shock proteins. Among the down-regulated proteins (Figure 6B), eleven are significantly related to ribosome biogenesis, including several 40S and 60S ribosomal proteins.

### 3.7. GC–MS Analysis of OAEO

The chemical composition of essential oil extracted from *O.* x *africanum* was analyzed by GC–MS. Twenty-five constituents were characterized and identified, which are presented in Table 3. The main constituents (having an area of more than 10%) were identified as 6-methyl-5-hepten-2-one (21.02%), citral (19.20%), neral (17.67%), and linalool (17.66%). Minor constituents (having 1–10% area) included α-pinene (5.99%), eucalyptol (2.28%), L-fenchone (2.14%), estragole (1.95%), 2,3-dehydro-1,8-cineole (1.46%), and α-terpineol (1.15%). The chromatogram of OAEO is shown in the Supplementary file (Figure S2, Table S1).

## 4. Discussion

Although *O.* x *africanum* has long been used in Thai traditional medicine, there is yet a lack of in vitro experiments exploring its activity against human cancers, including gastric cancer. We designed this study to investigate the in vitro anti-cancer activity of OAEO and the molecular mechanism by which it induces cell death in human gastric cells, specifically the AGS cell line. We first demonstrate that OAEO strongly inhibits AGS cell growth, with an IC_50_ of approximately 42.73 µg/mL, which is slightly higher than that of 5-FU (22.51 µg/mL); this finding suggests OAEO has anti-proliferative efficacy against AGS cells. Similarly, our previous data showed *O. tenuiflorum* essential oil (OTEO) to have in vitro growth-inhibiting effects and to induce apoptosis in the AGS cell line, with an IC_50_ of 163.42 µg/mL [12]. However, OAEO demonstrates a lower IC_50_ value and, hence, greater efficacy.

Apoptosis, known as programmed cell death, is a highly regulated physiological process that culminates in cell death and is integrally involved in the cellular development and homeostasis of animal tissues. Cancer cells engage diverse mechanisms to evade apoptosis, leading to their uncontrolled proliferation [23]. Apoptosis comprises two main pathways, termed the extrinsic and intrinsic apoptotic pathways [23]; both are downstream of the tumor suppressor p53, which is activated upon various cellular stresses and promotes either cell cycle arrest or apoptosis [24]. The extrinsic pathway is where the binding of death receptors, such as the type 1 TNF receptor or Fas receptor (CD95), activates caspase-8, an initiator caspase that cleaves other downstream (executioner) caspases [25]. In the intrinsic pathway, a group of proteins belonging to the Bcl-2 family (which comprises both anti- and pro-apoptotic proteins) disrupts mitochondrial membrane permeability and promotes the release of cytochrome c into the cytosol, where it forms the apoptosome that subsequently enhances the activation of caspase-9 [25]. In the final step of the process, the initiator caspase-8 or caspase-9 sets off the execution phase of apoptotic cell death by activating several downstream caspases, including caspase-3, which ultimately causes cytoskeleton reorganization, chromatin condensation, and chromosomal DNA fragmentation [23]. Previous reports have indicated that prolonged ER stress leads to the activation of caspase-12, which subsequently stimulates a cascade of caspase proteolysis that results in apoptotic execution [26,27].

The hallmark morphologic features of cells undergoing apoptosis, including cell shrinkage, membrane blebbing, and nuclear condensation/fragmentation [28], were observed in AGS cells exposed to OAEO for 6, 12, and 24 h. We also observed that the exposure of AGS to a pan-caspase inhibitor (z-VAD-fmk) before OAEO treatment resulted in a significant decrease in apoptotic cell death compared to OAEO treatment alone (data not shown), indicating that OAEO may induce apoptotic death in AGS cells via caspase activity. In addition, our qRT–PCR analysis showed that OAEO treatment resulted in the increased expression of *TP53* and the pro-apoptotic *BAX* gene, along with decreased expression of *BCL2* and *BCL-xL*, two genes responsible for inhibiting apoptosis and promoting aberrant cell proliferation. Simultaneously, OAEO treatment elevated the expression of *CASP9* and *CASP3,* while the expression of *CASP8* was not changed; these findings suggest that OAEO may induce apoptosis in AGS cells through the intrinsic mitochondrial pathway. We also observed that OAEO treatment stimulated the expression of *CASP12*; as caspase-12 activation is a recognized indicator of ER stress-induced apoptosis [26], this implies that OAEO-induced apoptosis may involve crosstalk between the ER stress and mitochondrial-apoptotic pathways. Relative gene expression of anti-apoptotic genes (*BCL2* and *BCL-xL*) was observed to be down-regulated in a time-dependent manner. However, in up-regulated pro-apoptotic genes (*TP53*, *BAX*, *CASP9*, *CASP12,* and *CASP3*), the relative expression was more after 12 h of treatment than at 24 h; this may be due to late apoptosis, with more dead cells at 24 h. While there is no prior evidence regarding the anti-cancer activity of *O.* x *africanum* specifically, extracts from related plants in the genus *Ocimum* have been reported to induce apoptosis. Ethanolic extracts or essential oils derived from *O. sanctum* or *O. basilicum* have been shown to effectively inhibit cell proliferation and induce apoptosis in several human cancer cell lines, including A549 lung adenocarcinoma cells [29], LNCaP prostate cancer cells [30], MCF-7 breast cancer cells [31], HeLa cervical cancer cells, and HEp-2 laryngeal epithelial carcinoma cells [32]. In addition, we previously demonstrated the ability of *O. tenuiflorum* essential oil to stimulate cell death in AGS cells via both extrinsic and intrinsic apoptotic pathways [12]. These previous findings support that compounds produced by *O.* x *africanum* may have potential use in strategies for cancer therapy.

To determine the early molecular events underlying OAEO-induced apoptosis in AGS cells, we used proteomic LC–MS/MS analysis to identify those proteins differentially expressed in the treated cells at 6 h of treatment due to the changes in cell morphology that began to appear. The most strongly up-regulated protein was 78-kDa glucose-regulated protein (GRP78), which has been characterized as a chaperone and stress sensor in the endoplasmic reticulum (ER); upon the development of ER stress, it is rapidly released from transmembrane signaling molecules and acts to bind and recover unfolded proteins [33,34]. The ER is an important subcellular organelle that is essential for intracellular homeostasis maintenance, with functions in controlling protein synthesis, the maturation and folding of secreted and membrane-bound proteins, and Ca^2+^ storage [5]. Several adverse physiological conditions, such as oxidative stress, hypoxia, and calcium depletion, can interfere with protein folding and result in the accumulation and aggregation of unfolded and misfolded proteins in the ER, which is recognized as ER stress [35]. When ER stress occurs, the unfolded protein response (UPR) is activated to relieve that stress [36], including through the release and activity of GRP78. The up-regulation of this protein in association with OAEO may reflect unfolded protein accumulation with consequent ER stress in treated AGS cells.

Heat shock proteins (HSPs) are chaperones implicated in a wide variety of cellular processes, including protecting the proteome from stress, promoting the correction of misfolded proteins, and directly re-folding misfolded proteins [37]. Thus, it has been proposed that the HSP response is activated by ER stress to relieve the stressful condition [38]. Indeed, our proteomic analysis revealed that the top five proteins most up-regulated by OAEO include 60 kDa heat shock protein (mitochondrial) (CH60), heat shock 70 kDa protein 1A/1B (HSP71), and heat shock 70 kDa protein 6 (HSP76), supporting the hypothesis that OAEO may induce ER stress by means of unfolded protein accumulation. Notably, when ER stress is sufficiently excessive or persistent, so as to overwhelm cellular protective mechanisms, apoptotic cell death is actively stimulated [39]. This implies that natural product agents that induce prolonged ER-stress-mediated apoptosis, such as OAEO, may suppress cancer cell growth and thus have important potential in therapeutic strategies for treating cancer.

In addition, the top five most down-regulated proteins in our proteome analysis of OAEO-treated AGS cells included 40S ribosomal protein S5 (RS5) and 60S ribosomal protein L31 (RL31). STRING analysis further revealed several ribosomal proteins as also down-regulated with OAEO treatment. Ribosome biogenesis is defined as the process of ribosomal RNA synthesis and processing; along with the assembly of rRNAs into complexes with ribosomal proteins, this is one of the most energetically demanding cellular activities [40]. Our findings suggest that OAEO induces the impairment of ribosome biogenesis, possibly by decreasing the function of enzymes involved in the critical energy-producing process of glycolysis, such as triosephosphate isomerase (TPI1). Perturbation of ribosome biogenesis is among the factors known to induce nucleolar stress, which ultimately results in the activation of p53 signaling and its downstream effects on cell cycle arrest, apoptosis, DNA repair, and so on [41]. Recent reports have indicated that several chemotherapy drugs used for treating hematological and solid malignancies are effective inhibitors of ribosome biogenesis at the level of rRNA transcription and/or processing [42].

In cancer cells, apoptosis can be triggered by oxidative stress caused by excess cellular levels of reactive oxygen species (ROS) [43]. However, cancer cells are well-adapted to withstand high levels of ROS and overcome ROS-induced cell death by up-regulating the production of endogenous anti-oxidant enzymes, thus protecting themselves from oxidative damage [44]. Peroxiredoxin-2 (PRDX2) has been identified as an endogenous peroxidase that functions to reduce oxidative stress and prevent apoptosis [45]. Previous studies have reported elevated PRDX2 expression in several types of cancer cells, including lung [46], liver [47], renal [48], and gastric carcinoma [49]. Interestingly, our proteome data revealed the down-regulation of PRDX2 in AGS cells after OAEO treatment. Jing et al. found that the knockdown of *PRDX2* in A549 non-small lung cancer cells promotes apoptosis by regulating the Bcl-2/BAX axis and caspase cascade [50]. In gastric cancer, patients with higher PRDX2 expression had significantly decreased survival compared to those with lower expression; additionally, in vitro knockdown of *PRDX2* significantly suppressed cancer cell proliferation and metastasis [49]. Based on our findings in the present study, we propose that OAEO treatment of AGS cells may generate oxidative stress while also attenuating PRDX2 expression, leading to severe oxidative stress, followed by the initiation of the ER stress condition and the impairment of ribosome biogenesis, ultimately mediating apoptotic cell death.

Metastasis refers to the ability of a cancer cell to disseminate from the primary tumor site to a distant organ. Aggressive carcinoma cells have been suggested to exhibit metastatic characteristics via a multistep process, including cell migration and invasion [51]. In addition to OAEO inducing cell death, we also demonstrated that it was able to markedly inhibit AGS cell migration in vitro. Transgelin-2 (TAGLN2) is an actin-binding protein that participates in the process of cytoskeleton remodeling [52]. Increased levels of transgelin-2 have been reported as associated with metastasis in several cancers, such as lung, bladder, colorectal, esophageal, and gastric cancer; moreover, its inhibition has negative effects on cancer cell migration and invasion [53]. Thus, this protein has been proposed to act as an oncogenic factor in a wide range of human malignancies. In the present study, we found that OAEO treatment of AGS cells significantly down-regulates transgelin-2 protein expression, which suggests that the essential oil’s ability to inhibit cell migration may be mediated by the down-regulation of transgelin-2. Importantly, transgelin-2 has previously been suggested as a molecular target protein that may have promising potential in cancer treatment [53].

Essential oils can be synthesized from diverse plant parts, including leaves, flowers, stems, bark, fruits, and roots, and comprise complex mixtures of organic volatile compounds, the vast majority of which belong to the terpene family [54]. Previous reports have suggested that many essential oils possess pharmacological properties, including anti-microbial, anti-oxidant, anti-inflammatory, and anti-cancer activities [55]. According to GC–MS analysis, we found the major constituents of OAEO to be 6-methyl-5-hepten-2-one, citral, neral, and linalool, while its minor constituents include α-pinene, eucalyptol, L-fenchone, estragole, 2,3-dehydro-1,8-cineole, and α-terpineol. This contrasts with a prior study conducted in Thailand that reported the essential oil from *O.* x *africanum* to contain neral and geranial as its major chemical components [10]. However, plant essential oils may vary in composition due to the influence of diverse exogenous and endogenous factors, including factors relating to the environment, the plant’s genetic characteristics, and its age [56]. We also expect that the chemical profiles of essential oils may be impacted by many other factors, such as abiotic and biotic environments, postharvest processing, the extraction process, and conservation conditions.

## 5. Conclusions

Taken together, the results from our study support that OAEO exerts anti-proliferative and anti-migration effects against the AGS human gastric cancer cell line. Applying both proteomic and molecular approaches, we conclude that OAEO-associated AGS cell death may be caused by the initiation of an oxidative stress condition that leads to ER stress and ribosome biogenesis impairment, followed by the induction of mitochondrial-apoptotic cell death. The observed in vitro anti-cancer activity of OAEO suggests that this essential oil may have the potential for further development as an alternative therapeutic approach for gastric cancer. However, additional studies are recommended to isolate and purify the individual constituents of OAEO, test their in vitro anti-cancer activities, and elucidate the underlying mechanisms of action. Moreover, to optimize its potential uses, the probable toxicology of OAEO should be further explored in an animal model.

## Figures and Tables

**Table 1:** Primer sequences used in qRT–PCR.

Gene	Primer Sequences	Annealing (°C)	Ref
*GAPDH*	5′-TCATCAGCAATGCCTCCTGCA-3′	55	[15]
	5′-TGGGTGGCAGTGATGGCA-3′		
*BCL-2*	5′-CAGGATAACGGAGGCTGGGATG-3′	60	[16]
	5′-AGAAATCAAACAGAGGCCGCA-3′		
*BCL-xL*	5′-ACCCCAGGGACAGCATATCA-3′	60	[17]
	5′-TGCGATCCGACTCACCAATA-3′		
*TP53*	5′-TAACAGTTCCTGCATGGGCGGC-3′	55	[18]
	5′-AGGACAGGCACAAACACGCACC-3′		
*BAX*	5′-TGGCAGCTGACATGTTTTCTGAC-3′	60	[19]
	5′-TCACCCAACCACCCTGGTCTT-3′		
*CASP8*	5′-AGAGTCTGTGCCCAAATCAAC-3′	60	[20]
	5′-GCTGCTTCTCTCTTTGCTGAA-3′		
*CASP9*	5′-CGAACTAACAGGCAAGCAGC-3′	60	[21]
	5′-ACCTCACCAAATCCTCCAGAAC-3′		
*CASP12*	5′-GCTCAGGAAATGGAAACAGC-3′	60	[22]
	5′-AGTGCTTGGTCCCACAGATT-3′		
*CASP3*	5′-GCGGTTGTAGAAGAGTTTCGTG-3′	60	[16]
	5′-CTCACGGCCTGGGATTTCAA-3′		

**Table 2:** List of top differentially expressed proteins (up and down) in AGS cells treated with OAEO.

Protein Accession	Protein Description	Fold Change	pI	% Coverage	Peptides Matched
GRP78_HUMAN	78 kDa glucose-regulated protein	+26.6	5.07	44.3	24
CH60_HUMAN	60 kDa heat shock protein, mitochondrial	+15.6	5.70	34.9	14
HSP71_HUMAN	Heat shock 70 kDa protein 1A/1B	+10.8	5.48	29	17
FLNB_HUMAN	Filamin-B	+10.00	5.49	23.5	45
HSP76_HUMAN	Heat shock 70 kDa protein 6	+8.8	5.81	20.5	12
RS5_HUMAN	40S ribosomal protein S5	−6.53	9.73	26.5	6
TAGL2_HUMAN	Transgelin-2	−5.97	8.41	82.4	16
RL31_HUMAN	60S ribosomal protein L31	−5.54	10.54	43.2	4
PRDX2_HUMAN	Peroxiredoxin-2	−5.13	5.66	25.3	5
TPIS_HUMAN	Triosephosphate isomerase	−3.90	6.45	56.2	13

**Table 3:** Chemical constituents of the essential oil from *O.* x *africanum*, analyzed by GC–MS.

Retention Time	Compound Name	CAS no.	Area (%)
2.37	Oxirane, tetramethyl-	5076-20-0	0.81
3.56	7-methyl-1,6-octadiene	42152-47-6	0.99
4.51	Pulegone	89-82-7	0.84
5.42	α-pinene	80-56-8	5.99
6.53	6-Methyl-5-hepten-2-one	110-93-0	21.02
6.75	2,3-dehydro-1,8-cineole	92760-25-3	1.46
7.05	3-hexen-1-ol, acetate, (e)-	3681-82-1	0.55
7.81	D-limonene	5989-27-5	0.76
7.91	Eucalyptol	470-82-6	2.28
9.01	Trans-linalool oxide (furanoid)	34995-77-2	0.54
9.56	L-fenchone	7787-20-4	2.14
9.89	Linalool	78-70-6	17.66
11.08	Photocitral A	55253-28-6	0.80
11.41	Trans-chrysanthemal	20104-05-6	0.89
12.92	α-terpineol	98-55-5	1.15
13.00	Estragole	140-67-0	1.95
13.87	β-myrcene	123-35-3	0.87
14.35	Neral	106-26-3	17.67
14.84	3-cyclohexen-1-one, 2-isopropyl-5-methyl	900155-47-0	0.50
15.32	Citral	5392-40-5	19.20
15.53	β-pinene	127-91-3	0.38
18.15	Camphene	79-92-5	0.14
18.77	Copaene	3856-25-5	0.29
20.16	Caryophyllene	87-44-5	0.56
20.55	Trans-α-bergamotene	13474-59-4	0.57

## Data Availability

Data sharing is not applicable.

## Supplementary Materials

The following supporting information can be downloaded at: https://www.mdpi.com/article/10.3390/medicina58060799/s1, Figure S1: Melting curve analysis of apoptosis-related genes in this study, Figure S2: The chromatogram of OAEO by GC–MS analysis, Table S1: Chemical constituents of the essential oil from *O.* x *africanum*, analyzed by GC–MS, Table S2: List of up-regulated proteins, Table S3: List of down-regulated proteins.
