# Supplementary material for: Endoplasmic Reticulum Stress and Impairment of Ribosome Biogenesis Mediate the Apoptosis Induced by Ocimum x africanum Essential Oil in a Human Gastric Cancer Cell Line
Source: Medicina (Kaunas). 2022 Jun 14;58(6):799. doi: 10.3390/medicina58060799 (PMC9227199; doi:10.3390/medicina58060799)
Supplement: Supplementary file 1 [file medicina-58-00799-s001.zip › medicina-1754849-supplementary.pdf]

Endoplasmic Reticulum Stress and Impairment of Ribosome Biogenesis Mediate the Apoptosis Induced by *Ocimum* x *africanum* Essential Oil ina Human Gastric Cancer Cell Line

Supplementary files

Figure S1

<table>
<tr><td>TP53</td><td>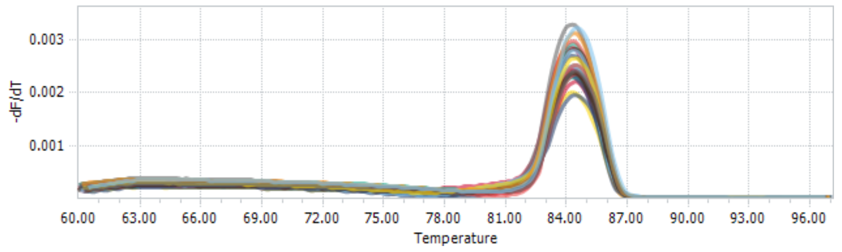
</td></tr>
<tr><td>BAX</td><td>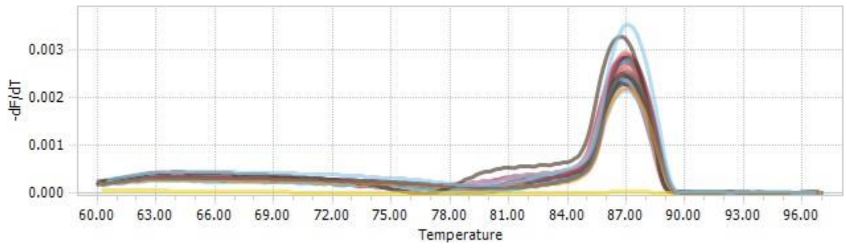
</td></tr>
<tr><td>BCL2</td><td>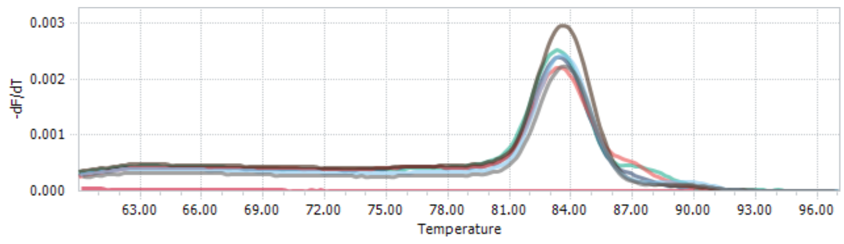
</td></tr>
<tr><td>BCL-xL</td><td>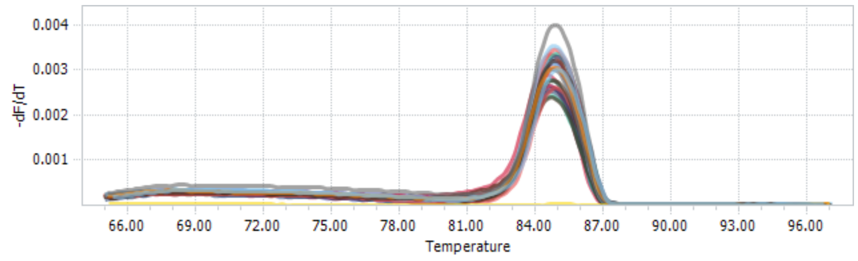
</td></tr>
</table>

CASP8	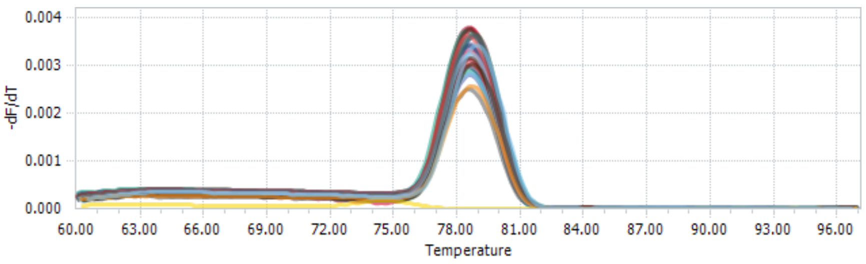0.004<br>0.003<br>0.002<br>0.001<br>0.000<br>-dF/dT<br>60.00 63.00 66.00 69.00 72.00 75.00 78.00 81.00 84.00 87.00 90.00 93.00 96.00<br>Temperature

CASP12

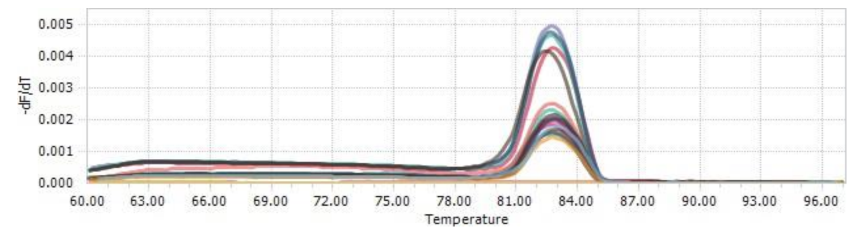

CASP9

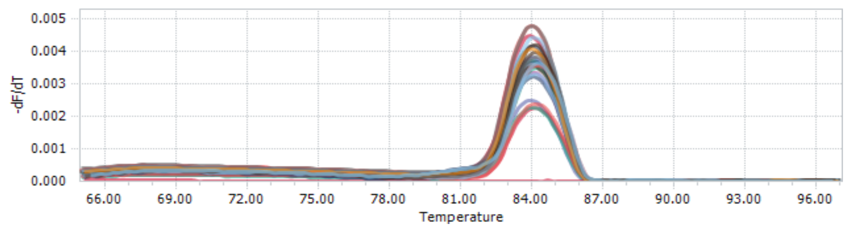

CASP3

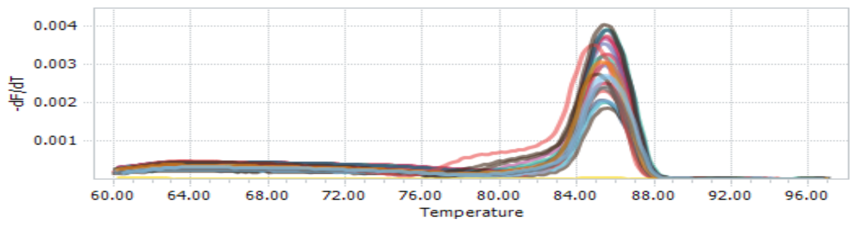

GAPDH

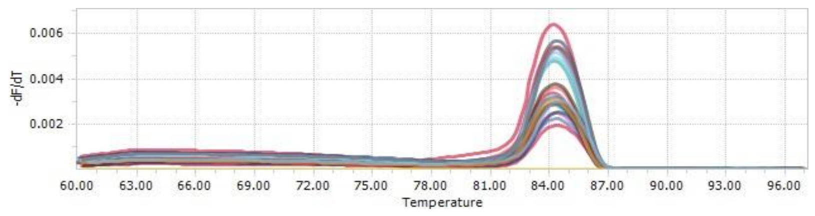

Figure S1. Melting curve analysis of apoptosis-related genes in this study.

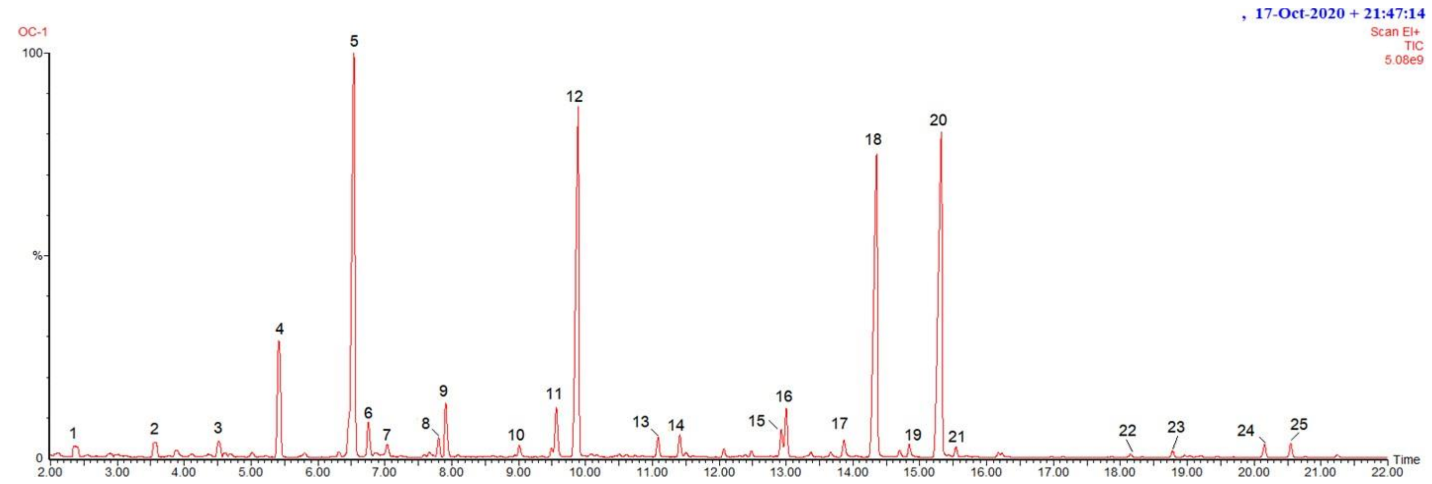

Figure S2. The chromatogram of OAEO by GC-MS analysis.

Table S1

Chemical constituents of the essential oil from *O.* x *africanum*, analyzed by GC-MS

No.	Quality	Retention time	Compound name	CAS NO.	Chemical structure	Area average	%
1	94.95	2.37	tetramethyl Oxirane	5076-20-0	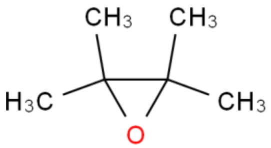	10986343	0.81
2	84.45	3.56	7-methyl-1,6-octadiene	42152-47-6	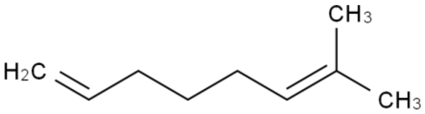	13448578	0.99
3	86.50	4.51	Pulegone	89-82-7	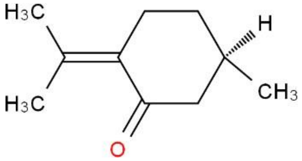	11474182.5	0.84
4	98.95	5.42	α-pinene	80-56-8	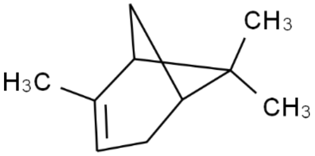	81397820	5.99
5	98.65	6.53	5-hepten-2-one, 6-methyl-	110-93-0	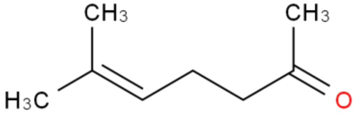	285837548	21.02
6	86.75	6.75	2,3-dehydro-1,8-cineole	92760-25-3	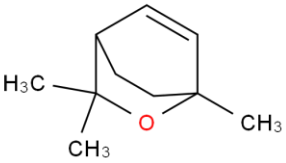	19813475	1.46

No.	Quality	Retention time	Compound name	CAS NO.	Chemical structure	Area average	%
7	97.35	7.05	3-hexen-1-ol, acetate, (e)-	3681-82-1	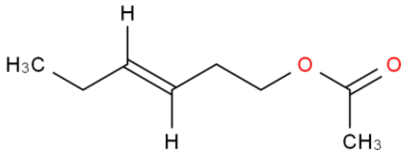	7459219	0.55
8	98.75	7.81	D-limonene	5989-27-5	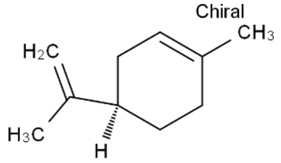	10275373	0.76
9	98.90	7.91	Eucalyptol	470-82-6	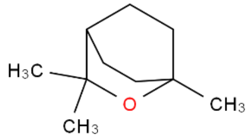	31025552	2.28
10	96.10	9.01	Trans-linalool oxide (furanoid)	34995-77-2	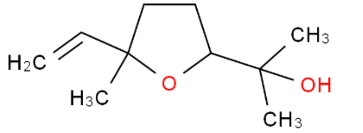	7279653	0.54
11	98.75	9.56	L-fenchone	7787-20-4	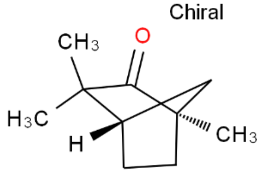	29133935.5	2.14
12	93.20	9.89	Linalool	78-70-6	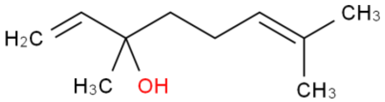	240195266.5	17.66
13	74.95	11.08	Photocitral A	55253-28-6	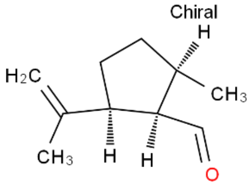	10928007.5	0.80

No.	Quality	Retention time	Compound name	CAS NO.	Chemical structure	Area average	%
14	86.55	11.41	Trans-chrysanthemal	20104-05-6	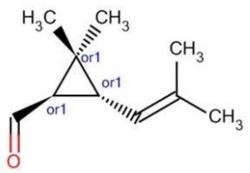	12090359	0.89
15	98.35	12.92	α-terpineol	98-55-5	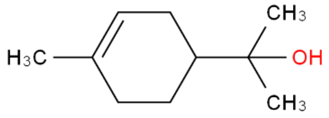	15687055	1.15
16	98.45	13.00	Estragole	140-67-0	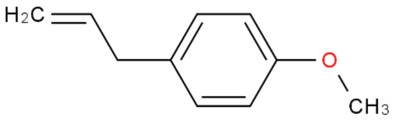	26457225.5	1.95
17	89.40	13.87	β-myrcene	123-35-3	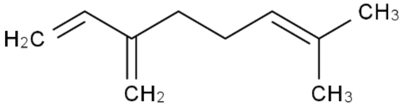	11841534	0.87
18	89.65	14.35	Neral	106-26-3	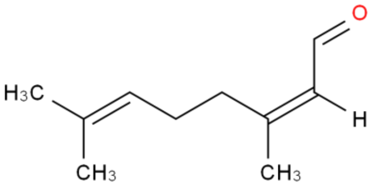	240318174.5	17.67
19	96.20	14.84	3-cyclohexen-1-one, 2-isopropyl-5-methyl	900155-47-0	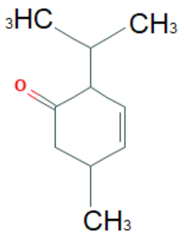	6861887	0.50

No.	Quality	Retention time	Compound name	CAS NO.	Chemical structure	Area average	%
20	97.15	15.32	Citral	5392-40-5	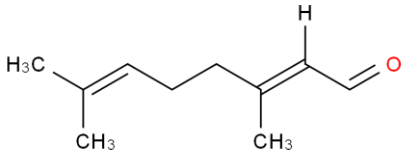	261080006.5	19.20
21	87.80	15.53	β-pinene	127-91-3	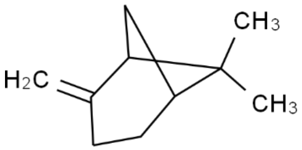	5207827	0.38
22	87.20	18.15	Camphene	79-92-5	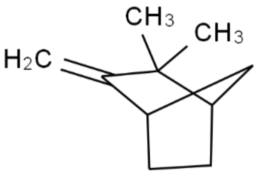	1903792	0.14
23	94.10	18.77	Copaene	3856-25-5	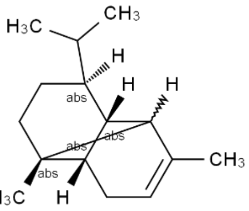	3931733	0.29
24	97.65	20.16	Caryophyllene	87-44-5	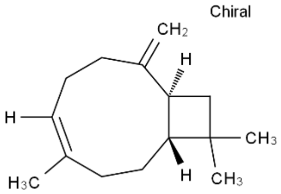	7625188	0.56
25	97.05	20.55	Trans-α-bergamotene	13474-59-4	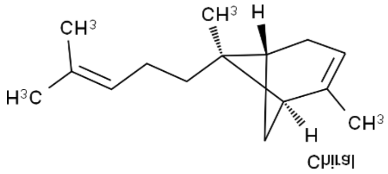	7732154	0.57

Gene Ontology (GO) enrichment analysis by STIRNG software

Parameters were set as shown below.

- Network Type: full STRING network
- Required score: medium confidence (0.400)
- FDR stringency: medium (5 percent)
- Organism: Homo sapiens

Gene Ontology (GO) enrichment analysis, Kyoto Encyclopedia of Genes and Genomes (KEGG)enrichment, Uniprot analyses were performed to predict the key pathways.

Table S2. List of up-regulated proteins

**prot_acc**	**prot_desc**	**SUM_Q**
GRP78_HUMAN	78 kDa glucose-regulated protein	26.6000
CH60_HUMAN	60 kDa heat shock protein, mitochondrial	15.6000
HSP71_HUMAN	Heat shock 70 kDa protein 1A/1B	10.8000
FLNB_HUMAN	Filamin-B	10.0000
HSP76_HUMAN	Heat shock 70 kDa protein 6	8.8000
EZRI_HUMAN	Ezrin	7.6000
HS71L_HUMAN	Heat shock 70 kDa protein 1-like	5.1000
FAS_HUMAN	Fatty acid synthase	4.5000
CLH1_HUMAN	Clathrin heavy chain 1	4.5000
TXND5_HUMAN	Thioredoxin domain-containing protein 5	4.4286
K1C18_HUMAN	Keratin, type I cytoskeletal 18	4.2143
HSP7C_HUMAN	Heat shock cognate 71 kDa protein	4.1818

SH3L3_HUMAN	SH3 domain-binding glutamic acid-rich-like protein 3	4.1212
K2C8_HUMAN	Keratin, type II cytoskeletal 8	4.0820
UBA1_HUMAN	Ubiquitin-like modifier-activating enzyme 1	4.0000
HSP72_HUMAN	Heat shock-related 70 kDa protein 2	3.8667
K22E_HUMAN	Keratin, type II cytoskeletal 2 epidermal	3.7561
ATPB_HUMAN	ATP synthase subunit beta, mitochondrial	3.3333
ACTN3_HUMAN	Alpha-actinin-3	3.3333
K2C7_HUMAN	Keratin, type II cytoskeletal 7	3.3333
K2C79_HUMAN	Keratin, type II cytoskeletal 79	3.0000
K2C73_HUMAN	Keratin, type II cytoskeletal 73	3.0000
K2C6B_HUMAN	Keratin, type II cytoskeletal 6B	2.6471
ACTB_HUMAN	Actin, cytoplasmic 1	2.6414
K2C5_HUMAN	Keratin, type II cytoskeletal 5	2.6364
ENPL_HUMAN	Endoplasmin	2.4737
CRIP1_HUMAN	Cysteine-rich protein 1	2.4146
ENOA_HUMAN	Alpha-enolase	2.2222
K2C75_HUMAN	Keratin, type II cytoskeletal 75	2.1818
CAP1_HUMAN	Adenylyl cyclase-associated protein 1	2.1667
GTR1_HUMAN	Solute carrier family 2, facilitated glucose transporter member 1	2.1667
RBM3_HUMAN	Putative RNA-binding protein 3	2.1500

OLA1_HUMAN	Obg-like ATPase 1	2.1429
NQO1_HUMAN	NAD(P)H dehydrogenase [quinone] 1	2.0909
AN32A_HUMAN	Acidic leucine-rich nuclear phosphoprotein 32 family member A	2.0833
AN32B_HUMAN	Acidic leucine-rich nuclear phosphoprotein 32 family member B	2.0833
EF1G_HUMAN	Elongation factor 1-gamma	2.0000
GANAB_HUMAN	Neutral alpha-glucosidase AB	2.0000
K22O_HUMAN	Keratin, type II cytoskeletal 2 oral	2.0000
KRT84_HUMAN	Keratin, type II cuticular Hb4	2.0000
K2C71_HUMAN	Keratin, type II cytoskeletal 71	2.0000
K2C3_HUMAN	Keratin, type II cytoskeletal 3	2.0000
K2C72_HUMAN	Keratin, type II cytoskeletal 72	2.0000

Table S3. List of down-regulated proteins

**prot_acc**	**prot_desc**	**SUM_Q**
RS5_HUMAN	40S ribosomal protein S5	6.5333
TAGL2_HUMAN	Transgelin-2	5.9703
RL31_HUMAN	60S ribosomal protein L31	5.5417
PRDX2_HUMAN	Peroxiredoxin-2	5.1333
TPIS_HUMAN	Triosephosphate isomerase	3.9000
RS19_HUMAN	40S ribosomal protein S19	3.6667
RS4X_HUMAN	40S ribosomal protein S4, X isoform	3.4545

IF6_HUMAN	Eukaryotic translation initiation factor 6	3.3077
PPIB_HUMAN	Peptidyl-prolyl cis-trans isomerase B	3.1667
1433Z_HUMAN	14-3-3 protein zeta/delta	3.1228
S10A6_HUMAN	Protein S100-A6	2.8101
H2A1B_HUMAN	Histone H2A type 1-B/E	2.5725
RS17_HUMAN	40S ribosomal protein S17	2.5000
PAL4B_HUMAN	Peptidylprolyl cis-trans isomerase A-like 4B	2.3659
LEG3_HUMAN	Galectin-3	2.2963
FKB1A_HUMAN	Peptidyl-prolyl cis-trans isomerase FKBP1A	2.2759
PRDX6_HUMAN	Peroxiredoxin-6	2.2308
RS20_HUMAN	40S ribosomal protein S20	2.2308
DEST_HUMAN	Destrin	2.2222
RL27_HUMAN	60S ribosomal protein L27	2.1818
RS16_HUMAN	40S ribosomal protein S16	2.1429
H90B3_HUMAN	Putative heat shock protein HSP 90-beta-3	2.1000
RL7A_HUMAN	60S ribosomal protein L7a	2.0909
RL18_HUMAN	60S ribosomal protein L18	2.0625
CAPZB_HUMAN	F-actin-capping protein subunit beta	2.0000
